# The Genitourinary Pathology Society and International Society of Urological Pathology Joint Expert Consultation Recommendations on intraductal carcinoma of the prostate

**DOI:** 10.1111/his.70046

**Published:** 2025-12-12

**Authors:** Rajal B Shah, Murali Varma, Ming Zhou, Gladell P Paner, Mahul B Amin, Daniel M Berney, Liang Cheng, Fang‐Ming Deng, Michelle Downes, Scott Eggener, Behfar Ehdaie, Jonathan I Epstein, Andrew Evans, Samson W Fine, Nancy Greenland, Charles Guo, Bo Han, Michelle S Hirsch, Kenneth A Izkowski, James G Kench, Tamara L Lotan, Cristina Magi‐Galluzzi, Hiroshi Miyamoto, Jane K Nguyen, Toyonori Tsuzuki, Theodorus H van der Kwast, Geert J van Leenders, Sean R Williamson, Sara E Wobker, Chin‐Lee Wu, Ximing Yang, Glen Kristiansen

**Affiliations:** ^1^ Department of Pathology University of Texas Southwestern Medical Center Dallas Texas USA; ^2^ Department of Cellular Pathology University Hospital of Wales Cardiff UK; ^3^ Department of Pathology, Molecular and Cell‐Based Medine Icahn School of Medicine at Mount Sinai New York New York USA; ^4^ Departments of Pathology and Surgery (Urology) University of Chicago Chicago Illinois USA; ^5^ Department of Pathology and Laboratory Medicine University of Tennessee Health Science System Memphis Tennessee USA; ^6^ Department of Urology USC Keck School of Medicine Los Angeles California USA; ^7^ Barts Health NHS Trust/Barts Cancer Institute Queen Mary University of London London UK; ^8^ Department of Pathology and Laboratory Medicine, Department of Surgery (Urology) Brown University Warren Alpert Medical School, the Legorreta Cancer Center at Brown University Providence Rhode Island USA; ^9^ Department of Pathology New York University Langone Hospital New York New York USA; ^10^ Precision Diagnostics and Therapeutics Program, Sunnybrook Health Sciences Centre University of Toronto Toronto Ontario Canada; ^11^ Departments of Urology University of California, Los Angeles (UCLA) Los Angeles California USA; ^12^ Department of Urology Memorial Sloan Kettering Cancer Center New York New York USA; ^13^ Integrated Medical Professionals Pathology Garden City New York USA; ^14^ Mackenzie Health Toronto Ontario Canada; ^15^ Department of Pathology and Laboratory Medicine Memorial Sloan Kettering Cancer Center New York New York USA; ^16^ University of California, San Francisco (UCSF) San Francisco California USA; ^17^ Department of Pathology The University of Texas MD Anderson Cancer Center Houston Texas USA; ^18^ BoQilu Hospital Shandong University Jinan China; ^19^ Department of Pathology Brigham and Women's Hospital and Harvard Medical School Boston Massachusetts USA; ^20^ Department of Pathology and Laboratory Medicine University of California – Davis Health Sacramento California USA; ^21^ Department of Tissue Pathology and Diagnostic Oncology NSW Health Pathology, Royal Prince Alfred Hospital Camperdown New South Wales Australia; ^22^ Brady Urological Institute and Department of Pathology Johns Hopkins University Baltimore Maryland USA; ^23^ Department of Pathology The University of Alabama at Birmingham Birmingham Alabama USA; ^24^ Department of Pathology and Laboratory Medicine University of Rochester Medical Center Rochester New York USA; ^25^ Robert J. Tomsich Institute of Pathology, Cleveland Clinic Cleveland Ohio USA; ^26^ Department of Surgical Pathology Aichi Medical University Nagakute Japan; ^27^ Laboratory Medicine Program, University Health Network and Princess Margaret Cancer Center University of Toronto Toronto Ontario Canada; ^28^ Department of Pathology Erasmus MC Cancer Institute Rotterdam The Netherlands; ^29^ University of North Carolina at Chapel Hill Chapel Hill North Carolina USA; ^30^ Department of Pathology Massachusetts General Hospital/Harvard Medical School Boston Massachusetts USA; ^31^ Northwestern University Feinberg School of Medicine Chicago Illinois USA; ^32^ Institute of Pathology University Hospital Bonn Bonn Germany

**Keywords:** AIP, atypical intraductal proliferation, IDCP, intraductal carcinoma, prostate cancer

## Abstract

Conflicting practice recommendations regarding the grading of intraductal carcinoma of the prostate (IDCP) from two leading uropathology societies, the Genitourinary Pathology Society (GUPS) and the International Society of Urological Pathology (ISUP), are confusing for both pathologists and treating clinicians. The objectives of this consultation were to clarify unresolved issues regarding IDCP and atypical intraductal proliferation (AIP) terminology, diagnostic criteria, grading, and management implications, as well as to develop uniform reporting guidelines for IDCP and AIP, endorsed by both societies. A 32‐member expert panel, composed of five core members, 25 expert urological pathologists, and two expert urologists, employed a modified Delphi process consisting of multiple rounds of consultation and voting. These were supplemented by discussions at the 2025 United States and Canadian Academy of Pathologists Annual Meeting to achieve expert consensus (defined as at least 67% agreement). Consensus was reached on several key issues. IDCP was regarded most commonly as reflecting the retrograde spread of invasive prostate cancer (PCa). IDCP diagnosis should be based on the Guo and Epstein criteria, supported by basal cell immunohistochemistry in cases that are difficult to distinguish from invasive PCa. The term AIP should be used only in equivocal proliferations where IDCP is favoured but the criteria are not fully met, and these should be reported as ‘*AIP, suspicious for IDCP*’. In the presence of invasive PCa, IDCP should generally be incorporated into Gleason grading irrespective of Grade Group (GG). However, a significant minority (30%) favoured excluding IDCP from the Gleason score if the invasive component was solely Gleason pattern (GP) 3. Pure IDCP (not associated with invasive PCa) and AIP, suspicious for IDCP, should not be graded. IDCP should not be incorporated in the grading of invasive PCa when it is spatially distinct from invasive PCa. A second opinion from a senior or dedicated GU pathologist and discussion within a multidisciplinary management setting should be considered, in the rare settings of pure IDCP or GP3 + IDCP (formerly GG1 + IDCP scenario). This joint GUPS–ISUP consultation provides unified recommendations for the diagnosis, terminology, grading, and reporting of IDCP and AIP, and will pave the way for the development of future IDCP/AIP WHO guidelines. Their adoption should reduce interobserver variation, facilitate consistent communication with clinicians, and improve patient management.

## Graphical abstract

The GUPS–ISUP joint expert consultation on IDCP provides unified recommendations for the diagnosis, terminology, grading, and reporting of IDCP and atypical intraductal proliferation. Their adoption should reduce interobserver variation, facilitate consistent communication with clinicians, and improve patient management.

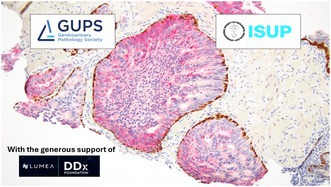

**Abbreviations**
AIP: atypical intraductal proliferation
GG: grade group
GP: Gleason pattern
GUPS: Genitourinary Pathology Society
HGPIN: high‐grade prostatic intraepithelial neoplasia
IDCP: intraductal carcinoma of the prostate
ISUP: International Society of Urological Pathology
NBX: needle biopsies
PCa: prostate cancer
PSA: prostate‐specific antigen
RP: radical prostatectomy

## Introduction

Intraductal carcinoma of the prostate (IDCP) is characterised as a lumen‐spanning proliferation of cytologically atypical prostatic secretory cells within distended pre‐existing ducts and acini, which displays at least partial preservation of basal cells. The concept of IDCP is not a new one, exquisitely illustrated by Hugh Hampton Young as early as 1909.^1^ In 1985, Kovi *et al*. showed that IDCP represents ‘ductal spread in prostatic adenocarcinoma’.^2^ A decade later, McNeal coined the term ‘IDCP’, and McNeal and Yemoto described diagnostic criteria for IDCP in radical prostatectomy (RP).^3^ IDCP gained wider recognition following a report by Guo and Epstein on pure IDCP in prostate needle biopsies (NBX), in which they established the diagnostic criteria for IDCP,^4^ which are currently most widely used in NBX and RP. In 2016, the WHO formally recognised IDCP as a distinct entity.^5^

IDCP in NBX can be encountered in two distinct scenarios: pure or isolated IDCP (not associated with an invasive component) and IDCP associated with invasive prostate cancer (PCa).^6^ Pure IDCP is rare, seen in <0.3% of NBXs,^7^ and is usually associated with unsampled, aggressive, invasive PCa.^8^ IDCP in RP specimens is generally associated with high‐grade invasive PCa but may rarely be seen in the absence of an invasive component or with only Grade Group (GG) 1 PCa. Hence, IDCP potentially may represent two distinct biological diseases: one that arises from a putative precursor (analogous to high‐grade prostatic intraepithelial neoplasia [HGPIN]) and a second that represents colonisation of high‐grade invasive PCa. Recently, the two major international genitourinary pathology societies (Genitourinary Pathology Society [GUPS] and International Society of Urological Pathology [ISUP]) published recommendations on grading PCa that included discussion on the reporting of IDCP. While the recommendations from the two groups were largely concordant, a significant difference was regarding the grading of IDCP.^9^, ^10^, ^11^ In the presence of invasive PCa, ISUP recommended that the IDCP component be incorporated into the Gleason Score (GS),^12^ whereas GUPS recommended reporting IDCP outside the GS with an explanatory comment.^13^ In most instances, IDCP is associated with high‐grade invasive PCa, and therefore, grading IDCP would not significantly change the GS, but there are several clinically relevant scenarios where grading IDCP would significantly change the reported GS. Although both societies recommend definitive therapy for patients with invasive PCa associated with IDCP diagnosed in NBX, differences in the reported GS could be confusing for clinicians and patients.^14^

Since HGPIN and IDCP may represent two ends of a morphological continuum, there would inevitably be intraductal proliferations with atypia greater than that seen in HGPIN but falling short of the diagnostic threshold for IDCP. Such proliferations have been referred to as atypical intraductal proliferation (AIP),^4^, ^15^, ^16^, ^17^ atypical cribriform lesion, borderline intraductal proliferation^18^, ^19^, atypical intraductal cribriform proliferation, and AIP, suspicious of intraductal carcinoma.^6^, ^16^, ^20^ ISUP and GUPS currently recommend the term AIP for these borderline intraductal proliferations that exhibit similar molecular and clinicopathologic characteristics as IDCP and may represent an under sampled IDCP.^20^, ^21^, ^22^

Despite significant progress in our understanding of IDCP, several issues remain controversial and unresolved about its terminology, diagnostic criteria, grading, and management. The conflicting practice recommendations from GUPS and ISUP are confusing for both pathologists and clinicians. ISUP and GUPS therefore organised a joint consultation which included expert pathologists from both societies, and two urologists in an advisory role. The objectives of this consultation were the following: (1) To clarify unresolved issues regarding IDCP and AIP terminology, diagnostic criteria, grading, and their management implications, and (2) to develop uniform reporting and grading guidelines for IDCP and AIP, endorsed by both societies.

## Materials and Methods

The Presidents of GUPS and ISUP (RBS and GK) invited three GU experts with documented expertise in IDCP to join them to form a 5‐member core group (RBS, GK, MZ, MV, and GPP) to lead this project. Following the discussion, this group decided to adopt a modified Delphi method (outlined below) to achieve expert consensus.

International genitourinary (GU) pathology guidelines have generally been developed by one of two approaches. In one approach, recommendations were based on voting results in consensus conferences attended by expert and non‐expert practicing pathologists, as well as some clinicians (urologists and oncologists).^12^, ^23^, ^24^ The participants voted on proposals outlined by working groups of invited experts. The second approach involves the construction of white papers by a small group of experts drafting a manuscript that was finalised following relatively unstructured discussions of comments and suggestions from a larger invited expert group, without any formal voting, to document the degree of expert consensus.^13^, ^25^ The evidence base in prostate pathology is inherently suboptimal due to the peculiarities of PCa biology, diagnosis, subjectivity, and management. In contrast to other tumours, many PCas are detected in non‐targeted biopsies that are subject to sampling inadequacies. Even more significantly, less than a third of localised PCas undergo RP, thereby allowing definitive assessment of pathological parameters such as tumour grade and pathological stage. The latter issue is particularly relevant when discussing IDCP, as it is often unclear whether IDCP in biopsies is associated with unsampled high‐grade invasive PCa. There is especially insufficient data in rare scenarios, such as IDCP that is not associated with invasive PCa or associated with only GG1 PCa. IDCP reporting recommendations are therefore generally based on subjective expert interpretation of inconclusive data. A modified Delphi approach with multiple rounds of consultation and voting was therefore adopted to canvass the opinions of the combined expert group. The process concluded with a formal vote on the final proposals to determine the degree of expert consensus on these sometimes complex and controversial issues. The Delphi process (method) that was originally designed for financial forecasting is based on the principle that structured group forecasts/decisions are more accurate than those from unstructured groups.^26^ The characteristic features of this process include anonymity, iterative feedback, group responses, and consultation on participants' opinions.

The Presidents of GUPS and ISUP invited 25 additional GU pathology experts and two urologists to join the core group to form a 32‐member expert panel. In total, RBS and GK each nominated 14 other pathologists and one urologist. The panel was selected based on clinical and research interests while ensuring geographic diversity. Voting was restricted to the pathologists, with the urologists playing an advisory role and providing a clinical context to the discussion. The core group designed and circulated a survey composed of 23 statements for the expert panel's consideration, together with short explanatory notes. The panellists voted to agree/disagree with these statements and were offered the opportunity to provide comments supporting their opinions. An anonymised summary of the voting results, together with a non‐anonymised collection of the comments, was circulated to the panellists, who had an opportunity to engage in the discussion by commenting on others' comments. Based on this discussion, some of the statements were refined for clarity, while others were split. Additional statements suggested by the group were also added. The modified version of the survey (29 statements) was recirculated to the group who voted to either agree or disagree (with the option to stay neutral by skipping a statement). These second‐round voting results were presented and discussed at a joint ISUP/GUPS consultation conference at the USCAP annual meeting in Boston on March 22, 2025, which was open to registered GUPS and ISUP members, with attendees offered an opportunity to comment on the statements. The conference meeting was attended by 216 participants. There was no voting during this meeting. After the Boston meeting, the statements were circulated along with all comments (including those at the consultation conference) for a third and final vote by the expert pathologists that forms the basis of the following discussion and recommendations. Consensus on a proposal statement was considered met when at least two‐thirds (67%) of the panel members agreed. Strong consensus was defined as ≥90% of the panel members agreeing.

## Results and Discussion

### Nature of IDCP

#### Background

IDCP, as defined by the stringent criteria proposed by Guo and Epstein,^4^ is usually associated with aggressive invasive PCa and is generally regarded as representing retrograde intraductal spread of invasive PCa.^27^, ^28^ However, rare cases of IDCP in RP specimens without invasive PCa or with only GG1 PCa have been reported.^29^ The latter has been interpreted as representing a putative precursor of PCa. Some experts have questioned the existence of precursor‐type IDCP because they have never encountered it in RP without invasive PCa^30^; however, in most centres, RP is performed only in patients with a preoperative histological diagnosis of invasive PCa. In contrast, a recent study using three‐dimensional microscopy concluded that IDCP is generally a pre‐invasive lesion with subsequent multifocal stromal invasion (repetitive invasion, precursor progression).^31^ However, these findings could also be explained by retrograde colonisation followed by multifocal ‘breakouts’ with stromal invasion by invasive‐type IDCP.^32^ Molecular data using next‐generation sequencing suggests that although the majority of IDCPs are clonally related to PCa, a minority of such proliferations are clonally distinct from PCa.^33^ This may be more frequent among cases of IDCP associated with GG1 cancer.^29^ While most cases of IDCP exhibit the cytomorphology of acinar PCa (intraductal acinar carcinoma), the ductal subtype of PCa may also retain a basal cell layer. The latter would represent intraductal spread of prostatic ductal adenocarcinoma (intraductal ductal carcinoma).^34^

#### Statements and voting results

The statements for all portions of the Delphi consensus, along with expert voting results, are summarised in Table 1.

**Table 1:** Statements and expert voting results

Statement #	Statement	# agree (%) *n* = 30	Conclusion
**Nature of IDCP**			
1	IDCP is biologically heterogeneous	30 (100%)	Total consensus in favour
2	Precursor‐type IDCP (definitively diagnosable only in prostatectomies) is a biological entity	28 (93%)	Strong consensus in favour
3	IDCP identified in biopsies is much more likely to represent invasive‐type IDCP than precursor‐type IDCP	28 (93%)	Strong consensus in favour
4	IDCP diagnosed with the modified Guo/Epstein criteria is generally associated with high‐grade invasive prostate cancer (sampled/unsampled)	30 (100%)	Total consensus in favour
5	In biopsies, GG1 with IDCP is generally associated with unsampled aggressive invasive prostate cancer	29 (97%)	Strong consensus in favour
**Diagnosis of IDCP**			
6	Diagnosis of IDCP should be based on Guo and Epstein criteria (or some variation of it)	28 (93%)	Strong consensus in favour
7	In rare cases, intraductal marked (pleomorphic/giant cell) atypia is sufficient for diagnosis of IDCP, even in the absence of expansile growth, solid or dense cribriform architecture or comedonecrosis	27 (90%)	Strong consensus in favour
8	AIP should be diagnosed only in proliferations in which IDCP is favoured (but criteria for IDCP are not met).	27 (90%)	Strong consensus in favour
9	Criteria for diagnosis of IDCP (all scenarios) should be loosened to include some AIP	7 (23%)	Consensus against
10	There should be separate criteria for the diagnosis of IDCP associated with invasive cancer and pure IDCP (not associated with invasive cancer)	2 (7%)	Strong consensus against
11	The number of loose cribriform atypical glands should be part of the IDCP diagnostic criteria	4 (13%)	Consensus against
12	Cytologically atypical cribriform proliferations in biopsies should reported as either AIP or IDCP (based on morphology)	30 (100%)	Total consensus in favour
13	ERG and PTEN immunohistochemistry can aid in the distinction of AIP from HGPIN	25 (83%)	Consensus in favour
14	Basal cell marker immunohistochemistry is recommended to distinguish AIP/IDCP from invasive cribriform pattern 4 prostate cancer when there is no obvious invasive cancer	30 (100%)	Total consensus in favour
**Terminology issues**			
15	From a pathobiological standpoint, the two types of IDCP should be referred to as precursor‐type IDCP and invasive‐type IDCP	25 (83%)	Consensus in favour
16	"AIP, suspicious for IDCP” is a better diagnostic term than “AIP"	3 (77%)	Consensus in favour
**Grading issues**			
17	Pure IDCP should not be graded	30 (100%)	Total consensus in favour
18	IDCP associated with GG ≥3 invasive cancer should be included in GS	23 (77%)	Consensus in favour
19	IDCP associated with GG2 invasive cancer should be included in GS	22 (73%)	Consensus in favour
20	In the presence GG1 invasive cancer, IDCP should be included in the overall grading with invasive cancer (grading based on the morphology of both invasive cancer and IDCP)	21 (70%)	Consensus in favour
21	In cases with invasive cancer and IDCP, there should NOT be different grading rules based on the grade of the invasive component	29 (97%)	Strong consensus in favour
22	IDCP clearly spatially distinct from invasive cancer (especially GG1) should not be included in the overall GS (included in a comment)	26 (87%)	Consensus in favour
23	Cribriform AIP associated with invasive cancer should be included in GS (grading based on H&E: cribriform morphology graded as pattern 4)	9 (30%)	Consensus against
24	There should be different rules for biopsies and prostatectomies regarding incorporation of IDCP in the GS	1 (3%)	Strong consensus against
**Management issues**			
25	Active surveillance is not appropriate for patients with GG1 associated with IDCP	28 (93%)	Strong consensus in favour
26	Management decisions for patients with isolated IDCP in biopsies should factor in MRI findings	27 (90%)	Consensus in favour
27	Management decisions for patients with isolated IDCP in biopsies should factor in morphology (whether IDCP appears morphologically invasive but basal cells present)	12 (40%)	No consensus
28	Management decisions for biopsy IDCP associated with invasive cancer should factor in MRI findings	25 (83%)	Consensus in favour
29	In rare scenarios such as pure IDCP and GG1 + IDCP diagnosed in prostate biopsies, consider obtaining a second opinion from a dedicated GU pathologist and an oncological urologist within a multidisciplinary setting	30 (100%)	Total consensus in favour

*n*, number of responses.

There was a strong consensus that IDCP is biologically heterogeneous, with precursor‐type IDCP (definitively diagnosable only in prostatectomies) representing a biological entity. There was unanimous agreement that IDCP diagnosed using the Guo and Epstein criteria is typically associated with sampled or unsampled high‐grade invasive PCa. There was also a strong consensus that IDCP identified in NBX is much more likely to represent invasive‐type IDCP than precursor‐type IDCP, and that NBX with GG1 invasive PCa and IDCP are generally associated with unsampled aggressive invasive PCa.

#### Discussion

During the consultation, it was noted that the precursor nature of IDCP identified in prostatectomy specimens without high‐grade invasive PCa cannot be definitively established in NBX specimens, as precursor‐type IDCP can be definitively diagnosed only after it has been excised. However, it is important to emphasise that there was complete (100%) consensus on the clinically significant point that IDCP is generally associated with high‐grade invasive PCa. The invasive PCa associated with IDCP is generally of high volume and represented in the biopsy samples, so pure IDCP is rarely encountered in clinical practice. Pure IDCP in biopsies is usually associated with aggressive invasive PCa in the unsampled prostate gland.

### Diagnosis of IDCP

#### Background

Diagnostic criteria for IDCP were first described by McNeal and Yemoto in 1996^3^ and refined for contemporary NBX specimens by Guo and Epstein in 2006.^4^ The Guo and Epstein criteria (Table 2) had some ambiguities that were clarified in the 2019 GUPS white paper on Gleason grading.^13^ The most notable change was the replacement of the specific size criterion (greater than 6x nuclear enlargement for cases lacking solid or dense cribriform morphology) with ‘bizarre pleomorphic cytological atypia’. It is important to note that the Guo and Epstein criteria were designed to identify patients with pure IDCP in NBX who could be recommended radical therapy even in the absence of associated invasive PCa because of the very high risk of unsampled aggressive invasive PCa.^4^ These criteria were very stringent and would have excluded IDCP at the lower end of the morphological spectrum to avoid the risk of overtreatment. Since AIP, diagnosed for lesions suspicious for, but not diagnostic for IDCP, shares clinical and molecular features with IDCP, and the management of pure IDCP in many institutions is early repeat NBX rather than radical therapy, some experts suggested that the diagnostic criteria for IDCP could be loosened to include some proliferations falling just short of the Guo and Epstein criteria. The Guo and Epstein criteria are currently also used to diagnose IDCP as an adverse prognostic parameter in NBX with invasive PCa. It is unclear whether these criteria are too stringent for use in this scenario and should be loosened when identifying IDCP associated with invasive PCa. Shah *et al*.^16^ reported that IDCP, in addition to Guo and Epstein IDCP criteria, were more likely to make up over six glands, gland >1 mm, and exhibit branching glandular contour, compared with cribriform HGPIN. Hence, it was suggested by some experts that the number of atypical cribriform glands, size, and glandular contour could help define the IDCP in some cases that do not otherwise meet the diagnostic threshold for diagnosis of IDCP; refining the criteria along these lines is an opportunity for further scholarship.

**Table 2:** Updated Guo and Epstein diagnostic criteria for IDCP

The specific nuclear size criterion (at least 6× normal) that was part of the original definition is not required
An intraductal proliferation of malignant prostatic epithelial cells with any of the following patterns
Solid pattern
2 Dense cribriform pattern (more cells than luminal spaces)
3 Loose cribriform/micropapillary pattern with either of the following
Marked (bizarre/pleomorphic) nuclear atypia
b Non‐focal comedonecrosis

**Figure 1:**
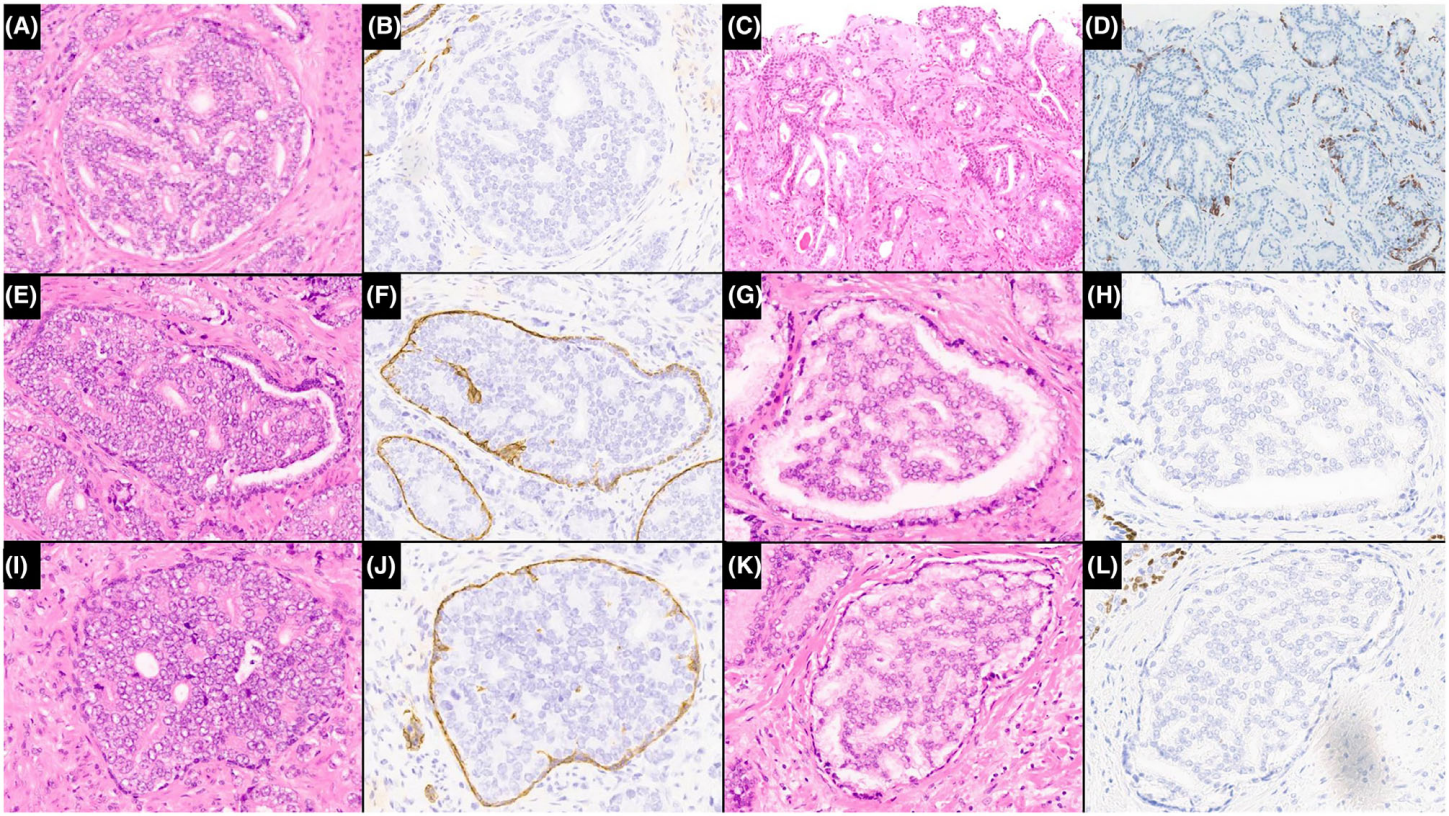
There is a morphological overlap between IDCP and invasive adenocarcinoma. Like IDCP, invasive cancer may have rounded outlines (**A, B**) while IDCP may show irregular contours (**C, D**). Prostate cancer (**E, F**) can mimic partial involvement of benign glands by IDCP (**G, H**). Flattened peripheral tumour cells in prostate cancer may mimic basal cells (**I, J**), thereby resembling the basal cell layer of IDCP (**K, L**) [**B, D, H, L**: CK 5/6; and **F, J**: p63]. Reproduced with permission from Varma.^6^

**Figure 2:**
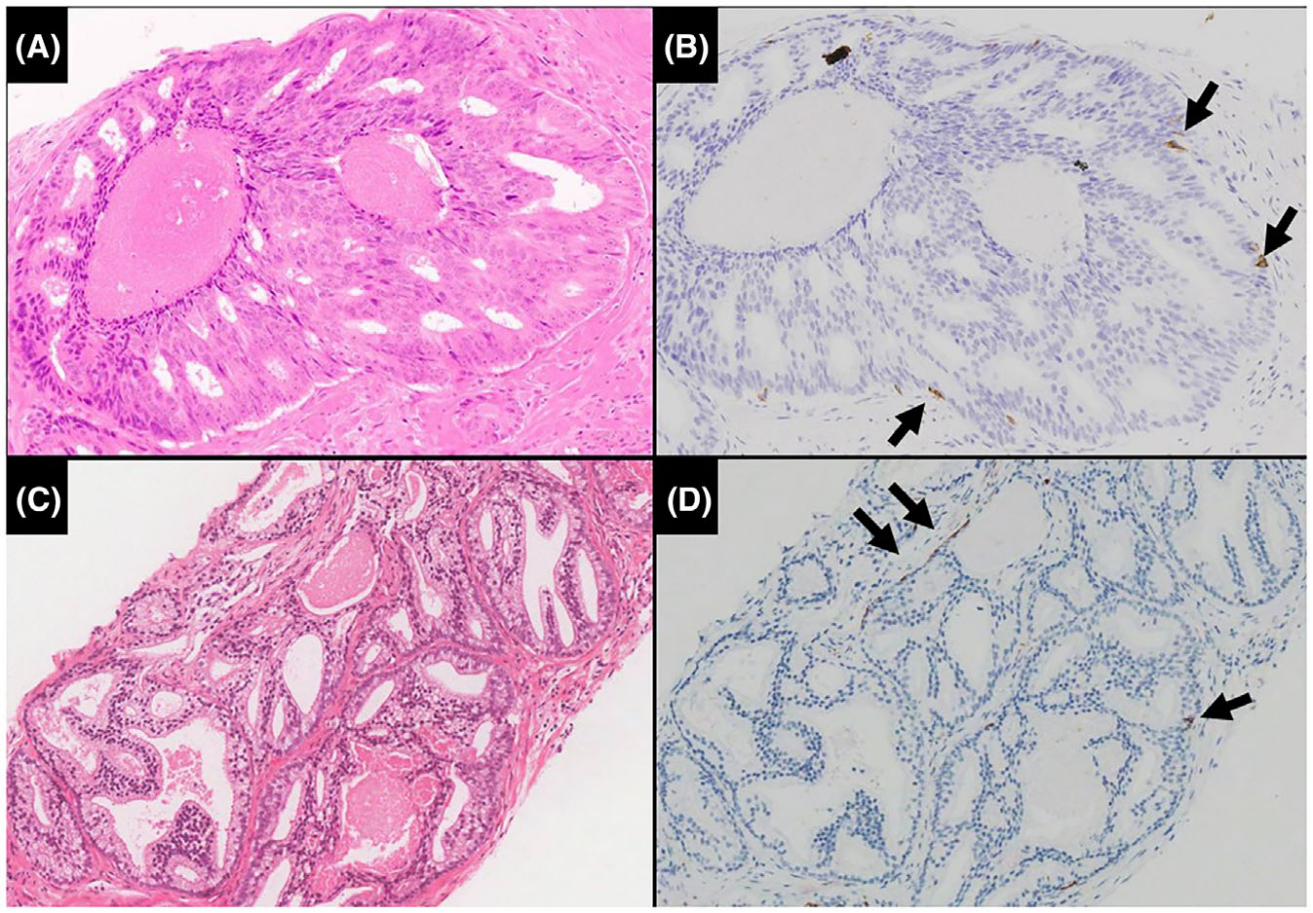
There is an immunohistochemical overlap between IDCP and invasive adenocarcinoma. The basal cell layer is often discontinuous (i.e., scattered basal cells present) in IDCP, so basal cells may not be apparent in the tissue planes examined (**B, D**: CK 5/6). Reproduced with permission from Varma.^6^

In addition to these morphological criteria, immunohistochemistry may aid in the diagnosis of IDCP, specifically the distinction from invasive PCa. Retention of the basal cell layer is a prerequisite for IDCP, but it can be difficult in a subset of cases to definitively identify basal cells in H&E‐stained sections, as these can be closely mimicked by flattened peripheral tumour cells (Figure 1). Conversely, the absence of basal cells would not definitively exclude the possibility of IDCP, as the basal cell layer can be discontinuous and sparse and therefore may be out of the plane of section (Figure 2).^35^, ^36^, ^37^ ERG and PTEN immunohistochemistry may also help distinguish IDCP from HGPIN. HGPIN is typically ERG negative/PTEN intact, while IDCP may be ERG‐positive/PTEN loss in ~ 50% of cases,^18^, ^19^ as ERG positivity and PTEN loss are not seen in all invasive PCas (also ~50% of cases). Hence, while ERG‐positive/PTEN loss immunoprofile would favour IDCP, the converse immunoprofile (ERG‐negative/PTEN intact) would not exclude the possibility of IDCP. ERG‐positive/PTEN loss immunoprofile would also not distinguish between IDCP and AIP.^15^, ^18^, ^19^, ^20^ There are also limited data on racial differences in the positivity for ERG, and as such, all immunohistochemical data should be evaluated strictly in the context of morphology and with clinical implications being kept in mind.^38^, ^39^

#### Statements and voting results

There was strong consensus that the diagnosis of IDCP should be based on the modified Guo and Epstein criteria (Table 2) and that in rare instances, marked (bizarre/pleomorphic) intraductal cytological atypia alone is sufficient for the diagnosis of IDCP. There was also strong consensus against lowering the threshold for the diagnosis of IDCP in cases with associated invasive PCa, and also against using the number of loose cribriform atypical glands to diagnose IDCP. Finally, there was consensus against loosening the diagnostic criteria for pure IDCP in NBX to include some cases at the more atypical end of the AIP spectrum. There was consensus that ERG and PTEN immunohistochemistry, if available, may aid in the diagnosis of AIP/IDCP and its distinction from HGPIN. There was a strong consensus to recommend the use of basal cell marker immunohistochemistry to distinguish AIP/IDCP from invasive cribriform pattern 4 PCa when there is no obvious invasive PCa.

#### Discussion

**Figure 3:**
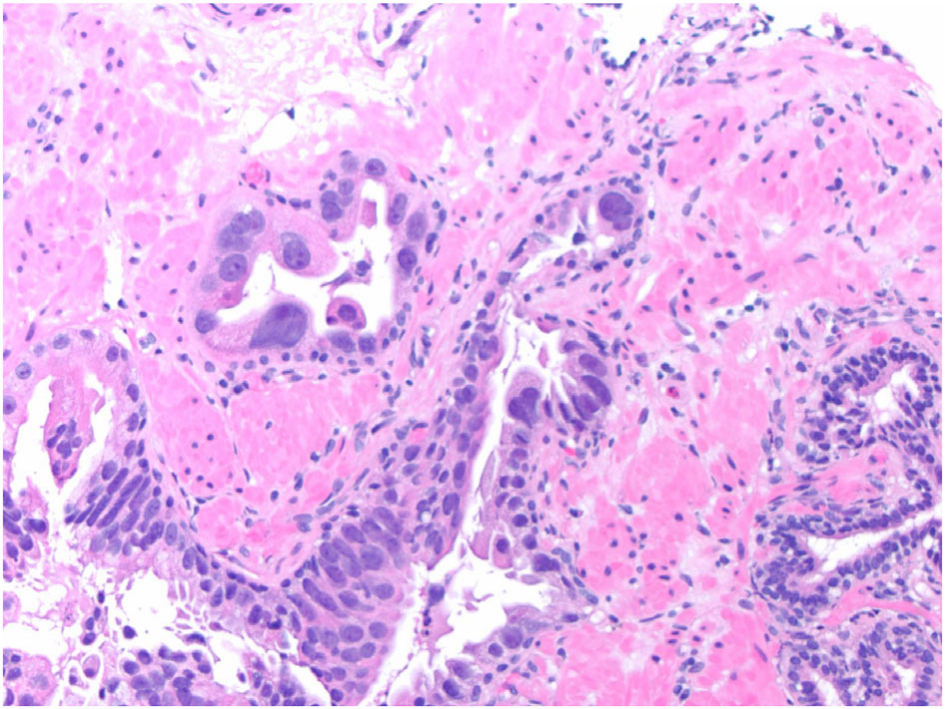
IDCP, diagnosed based solely on the presence of bizarre/pleomorphic cytological atypia. In such a scenario, radiation treatment‐related cytological atypia and/or colonisation by high‐grade urothelial carcinoma should be excluded.

Some experts expressed reservations regarding some aspects of the Guo and Epstein criteria for IDCP, particularly the marked nuclear pleomorphism criterion that is rarely encountered in isolation, with little available outcome data for this scenario. However, the consensus was that in rare cases, intraductal marked (bizarre/pleomorphic) atypia is sufficient for the diagnosis of IDCP, even in the absence of expansile growth, solid or dense cribriform architecture, or comedonecrosis. It is important to emphasise that this is an extremely rare scenario and would require truly bizarre cytological atypia analogous to that seen in pleomorphic giant cell adenocarcinoma of the prostate (Figure 3). In this situation, it is also important to exclude radiation treatment‐induced cytological atypia and spread of high‐grade urothelial carcinoma before making the diagnosis of IDCP. Many experts felt that the Guo and Epstein criteria may be too stringent for diagnosing IDCP associated with invasive PCa, but there was a strong consensus that having separate criteria for the diagnosis of IDCP associated with invasive PCa and pure IDCP (not associated with invasive PCa) would be too confusing for surgical pathologists. However, diagnostic criteria such as estimation of the degree of cellular density (loose versus dense cribriform) and nuclear atypia are subjective, and it may be reasonable to ‘err on the side of’ IDCP in truly borderline cases if there is associated invasive PCa. However, it would be important to retain a high diagnostic threshold for pure IDCP in biopsies if this diagnosis could trigger radical therapy despite the absence of an identified invasive component. A high diagnostic threshold is also recommended for IDCP associated with GG1 invasive PCa because this diagnosis could preclude active surveillance. Many experts would consider the number of atypical glands when distinguishing AIP from IDCP and tend towards the latter diagnosis when several atypical glands with morphological features bordering on IDCP are present, particularly when these are associated with invasive PCa. However, it is unclear what the cut‐off number should be, and there was a strong consensus among this group that this should not be part of the diagnostic criteria for IDCP. A diagnosis of IDCP can be rendered if even a single gland shows unequivocal features of IDCP.

Flattened peripheral tumour cells can closely mimic basal cells on morphological examination (Figure 1). Hence, there was complete consensus that the diagnosis of pure IDCP in NBX should be confirmed with basal cell marker immunohistochemistry. Basal cell immunohistochemistry may also be required in the setting when IDCP is associated only with GP3 PCa. A rare exception to this rule could be partial colonisation of benign glands by IDCP. ERG‐positive/PTEN loss immunoprofile would support a diagnosis of AIP/IDCP over HGPIN. However, as indicated earlier, IDCP may be ERG‐negative/PTEN intact, so ERG and PTEN immunohistochemistry are not recommended in intraductal proliferations that meet the morphological diagnostic criteria for IDCP.

### Diagnosis of AIP

Even though HGPIN and IDCP may be clonally distinct, they often represent a morphological continuum, so there is inevitably a grey zone between these putative biological entities. Some intraductal proliferations show a greater degree of cytological/architectural atypia than that typically seen in HGPIN, but do not meet the stringent Guo and Epstein criteria for the diagnosis of IDCP. Such proliferations have been termed ‘AIP’ and generally trigger repeat NBX to exclude IDCP. Repeat NBX is an invasive procedure with significant potential morbidity and may not be appropriate for proliferations at the lower end of the AIP spectrum, where HGPIN is favoured, but the possibility of IDCP cannot be confidently excluded. In this setting, an ERG‐positive/PTEN loss immunoprofile, if available, may be helpful in distinguishing from HGPIN, but an ERG‐negative/PTEN intact immunoprofile would not be helpful. In this scenario, imaging and other biomarker testing, such as prostate‐specific antigen (PSA) monitoring, as would be the norm following a HGPIN diagnosis, could be sufficient, and diagnosis of AIP restricted to equivocal proliferations in which IDCP is favoured.

#### Statements and voting results

It was proposed that ‘AIP’ should be diagnosed only in proliferations in which IDCP is favoured (but the criteria for IDCP are not definitively met). There was a strong (90%) consensus in favour of this proposal.

#### Discussion

It is important to avoid overdiagnosis of AIP, which could result in unnecessary invasive investigation. Underdiagnosis of AIP (as benign or HGPIN) at the lower end of the morphological spectrum is less critical, as these patients would likely undergo PSA monitoring with repeat NBX that would identify clinically significant PCa without an excessive delay. The recommendation to restrict the diagnosis of AIP to proliferations suspicious for IDCP would have ramifications for reporting terminology that is covered in the next section.

### Terminology Issues

#### AIP

The term ‘atypical intraductal proliferation’ is potentially ambiguous since proliferations such as HGPIN are also composed of an intraductal proliferation of atypical prostatic epithelial cells. As indicated above, there was a strong consensus among this expert group that the diagnosis of AIP should be restricted to proliferations in which IDCP is favoured (suspicious for IDCP). WHO 2022 defines AIP as ‘an atypical intraductal proliferation characterized by morphologic features exceeding that of HGPIN but not meeting strict diagnostic criteria for IDCP’.^5^ An issue with this definition is that it could be reasonably interpreted by practicing pathologists to include proliferations towards the lower end of the grey zone in which HGPIN is favoured, but the possibility of IDCP cannot be excluded.

#### Statements and voting results

It was proposed that ‘*AIP, suspicious for IDCP'*, is a better diagnostic term than *AIP* alone. There was a 77% consensus in favour of this proposal.

#### Discussion

It is recommended that a diagnosis of AIP should be rendered only in proliferations suspicious for IDCP and that this should be effectively communicated by using the term ‘*AIP, suspicious for IDCP*’. An explanatory comment would not be required if this unambiguous diagnostic terminology is used. However, there would inevitably be proliferations at the lower end of the spectrum in which the pathologist, while not suspicious for IDCP, is uncomfortable reporting as HGPIN or benign. Any AIP with cribriform architecture should disqualify the diagnosis of HGPIN and should be reported as *AIP, suspicious for IDCP*. ERG and PTEN immunohistochemistry, if available, may be useful in a subset of AIP bordering on HGPIN. ERG‐positive/PTEN loss would favour *AIP, suspicious for IDCP*.

#### IDCP

As indicated earlier, there was strong consensus among this group that IDCP is heterogeneous and that while it generally represents intraductal spread of high‐grade invasive PCa, a minority view was that this non‐invasive proliferation mostly represents a putative precursor of PCa. It is important to note that only the putative precursor would be considered a distinct biological entity, as the common form of IDCP would represent a growth pattern of invasive PCa (acinar or ductal type). However, the term IDCP describes the morphology rather than the biology of the two types of intraductal proliferation. For example, in the 2022 WHO blue book, the section on IDCP describes an intraductal proliferation with an in situ behaviour code of a ductal carcinoma (M8500/2), with most of the accompanying text referring to intraductal spread of invasive acinar PCa (M8040/3).^27^, ^33^ Also, the use of the term IDCP could be ambiguous, so separate terms for the two types of IDCP (precursor‐type IDCP and invasive‐type IDCP) were proposed. As it is generally not possible to morphologically distinguish these two forms of IDCP in prostate NBXs, the proposed terms (precursor‐type IDCP and invasive‐type IDCP) should not be used as diagnostic terminology in histopathology reports, particularly in the biopsy setting.

#### Statements and voting results

It was proposed that ‘from a pathobiological standpoint (e.g., research, academic discussions), the two types of IDCP should be referred to as *precursor‐type IDCP* and *invasive‐type IDCP*’ and that these two terms would not be used in diagnostic reports. There was an 83% consensus in favour of this proposal.

#### Discussion

It is emphasised that these terms were not proposed as diagnostic entities for use in histopathology reports, but only from a conceptual standpoint to make clear which type of IDCP is being discussed. The recommended diagnostic terminology for biopsy reports would be the generic *IDCP*. However, these terms could be used in the comment section to clarify the differential diagnosis. For example, in NBX with IDCP with GG1 PCa, the following explanatory note could be added: ‘Such cases are generally associated with unsampled high‐grade invasive prostate cancer, but the possibility of so‐called precursor‐type IDCP cannot be excluded’.

### Grading IDCP

#### Background

Due to the cribriform nature of most of the IDCP, including IDCP in the GS would often increase the GP4 component in the GS. There is a general consensus in the literature that pure IDCP should not be graded.^12^, ^13^, ^24^ However, there is significant controversy about whether IDCP associated with invasive PCa should be included in the GS, with conflicting recommendations published by GUPS and ISUP.^12^, ^13^ The arguments in favour and against the incorporation of an IDCP component into the GS have been detailed in a recent debate.^40^

IDCP is generally associated with high‐grade invasive PCa, so this variation in reporting practice would affect the reported GG in only a subset of patients.^41^, ^42^ The impact on NCCN risk stratification could be even less, as many of these patients would have other adverse prognostic factors, such as high PSA or high clinical stage.^43^ However, these two different grading methods could lead to the reporting of significantly different GSs in some patients, which in turn could result in significantly different treatment recommendations. Moreover, this difference in reporting practice can cause significant confusion for clinicians and patients, particularly since reports do not often indicate which rules have been followed.

The main argument in favour of excluding IDCP from the GS is that in a minority of patients, this component could represent precursor‐type IDCP that should not be graded. Incorporating IDCP into the GS could result in over‐grading of these patients, particularly when the invasive component is purely GP3. In the only published series of patients with NBX GG1 + IDCP who underwent RP, the invasive PCa in the prostatectomy specimen was GG1 in 3 (21%) of 14 patients.^44^ However, in all three of these low‐grade (GG1) cases, the RP had been only partially submitted for histological examination, and one of these patients developed biochemical recurrence.

The primary rationale of including IDCP in the GS is that, in the vast majority of patients, this component represents invasive‐type IDCP. Thus, most patients with NBX GG1 and IDCP would have unsampled high‐grade invasive PCa, resulting in an adverse outcome. It is noteworthy that in the aforementioned study of biopsy GG1 + IDCP,^36^ 12 (19%) of 62 men with available follow‐up data developed bone metastasis during the follow‐up period. Accordingly, if the IDCP component is excluded from PCa grade, then the reported GS may underestimate patient risk with a potential for undertreatment if the included comment is not correctly interpreted by the treating clinician. Other issues with excluding IDCP from the GS include the difficulty in distinguishing IDCP from invasive PCa by morphology and even immunohistochemistry [Figures 1 and 2], and explanatory comments may not be adequately represented in epidemiological databases.

In rare cases, IDCP may be present in biopsies that are clearly spatially distinct from invasive PCa. For example, cores from the right side may show invasive PCa while IDCP is seen only in a contralateral biopsy. If separate GSs are reported from each biopsy/specimen, then a GS would be reported only for the invasive PCa, with the other core reported as pure IDCP (i.e. neither graded itself, nor included in grade). However, if an overall (i.e., global) GS is reported for a set of prostate NBXs, guidance on whether or not the IDCP component should be incorporated into this overall GS should be provided.

The clinical significance of grading (or not grading) the IDCP component of invasive PCa also depends on specimen type and the grade of the associated invasive PCa. The impact would be less in NBXs with high‐grade invasive PCa and in RP specimens, where the GS is less likely to affect patient management. Hence, the possibility of having different rules regarding IDCP grading for NBX and RP, as well as in NBXs based on the grade of the invasive component, was explored. The primary objection to not recommending having different rules for different specimens/scenarios is that this would be too confusing for practicing pathologists.

The Gleason grading system is based on morphology, so morphologically invasive, loose cribriform glands with retained basal cell layer that do not meet the stringent IDCP criteria (AIP, suspicious for IDCP) associated with invasive PCa may traditionally have been included in the GS. Studies have demonstrated that AIP, suspicious for IDCP, is a marker for unsampled IDCP and has an outcome similar to IDCP.^15^, ^16^, ^22^ AIP, suspicious for IDCP and IDCP also share many molecular characteristics.^15^ Hence, it has been argued that AIP, suspicious for IDCP associated with invasive PCa should be included in the GS if morphologically compatible with invasive PCa.^32^ However, AIP, suspicious for IDCP is a descriptive diagnosis, and since it is not an entity without definitive diagnostic criteria, it must not be included in the GS.

#### Statements and voting results

There is complete consensus that pure IDCP should not be graded, and there is a consensus to grade IDCP in the presence of PCa of any GG, although a significant minority (30%) favoured excluding IDCP from the GS, particularly when the invasive component was solely Gleason pattern 3. There is a strong consensus against applying different grading rules regarding IDCP grading in NBX and RP specimens, as well as in cases with PCa of different GG. There is consensus that IDCP that is spatially distinct from invasive PCa should not be included in the GS and that AIP associated with invasive PCa should also not be included in the grade.

#### Discussion

The consensus opinion of this joint GUPS/ISUP expert group was that while pure IDCP should not be graded, the IDCP component associated with invasive PCa of any grade and in any specimen should be included in the GS unless clearly spatially distinct (for example, in contralateral NBX). Grading of the IDCP component would be based on morphology (cribriform: pattern 4, solid/comedonecrosis: pattern 5). In rare cases with only marked (bizarre/pleomorphic) cytological atypia, until more evidence is available, the IDCP component should not be included in the final grade.

The presence of an associated IDCP component should be commented on after incorporating it into the GS, as some studies suggest that the presence of IDCP may predict resistance to some therapies^45^, ^46^ and may be indicative of a *worse* prognosis than invasive PCa of corresponding morphology.^47^, ^48^ However, these studies require further independent validation, and it is unclear whether the treatment resistance associated with IDCP is greater than that of invasive PCa of corresponding morphology. A comment to indicate the presence of IDCP is also particularly critical for patients with pure GP3 and IDCP, since these would be graded as GG2 when IDCP is incorporated, and could be falsely assumed to be eligible for active surveillance. Additionally, stating the presence of IDCP in pathology reports (even when used as part of the Gleason score) is important for genetic testing, as this is recommended by NCCN and other similar guidelines.^49^, ^50^

IDCP can be difficult to distinguish from invasive PCa by morphology in a subset of cases [Figure 1], so immunohistochemistry is often required to confirm the presence/absence of basal cells. If IDCP is incorporated into the GS, then its identification in the setting of a definite invasive component is generally less important. However, in the setting of a definite invasive component, the use of immunohistochemistry to distinguish IDCP from invasive PCa may still be needed in the rare cases when the IDCP component is included with GP3 to determine grade or in line with local institutional practice, where management decisions are based on the presence/absence of IDCP distinct from cribriform/comedonecrosis invasive PCa.

### Management of IDCP

#### Background

It was envisioned that this consultation would be able to optimise and standardise the pathologists' approaches in diagnosing and grading IDCP for enhanced prognostic information that can inform future clinical management guidelines. In addition, another objective was to convey expert pathologists' opinions on management issues that could help clinicians formulate their recommendations. Clinicians acknowledge IDCP to be an unfavourable histology, and some clinical guidelines recommend that patients with IDCP in biopsy should not be placed on the active surveillance protocol.^51^, ^52^ However, IDCP is currently not incorporated in contemporary PCa risk stratifications used in management algorithms.^52^, ^53^ The prevalence of IDCP increases from 2.1% in low‐risk patient cohorts to 23.1%, 36.7%, and 56% in moderate‐risk, high‐risk, and metastatic or recurrent disease risk categories, respectively.^45^ Grade is an established determinant of PCa risk, but the incorporation of IDCP into the grading of invasive PCa has been debated, resulting in a lack of uniformity in grading^10^, ^12^, ^13^; this is a major issue that is now addressed through this consultation. IDCP is typically associated with higher grade and stage PCas, and thus, it is expected that the proportion of IDCP increases with increasing PI‐RADS scores on multiparametric magnetic resonance imaging (mpMRI).^54^, ^55^ Overall, mpMRI shows promising sensitivity but only moderate specificity in detecting cribriform architecture, including IDCP.^54^, ^56^ Large prospective studies are still needed to validate these radiologic findings.

#### Statements and voting results

There was a strong consensus among experts that the current practice of active surveillance is not appropriate for patients with GG1 PCa associated with IDCP. Due to the rarity and uncertainty in managing these cases, there was a unanimous agreement that cases of GG1 PCa associated with IDCP should be reviewed by a pathologist with GU pathology expertise and discussed in a multidisciplinary setting. There was a strong consensus that management decisions for patients with either pure IDCP or IDCP associated with invasive PCa should factor in MRI findings.

#### Discussion

Prevailing clinical practices suggest that IDCP may affect management decisions when IDCP is present in pure form or if associated with GG1 PCa. A recent GUPS survey among clinicians showed that 76% of urologists and 59% of radiation oncologists will exclude patients with IDCP from active surveillance protocols. In contrast, 71% of urologists and 66% of radiation oncologists indicated that the presence of IDCP in GG2‐5 PCa will not affect their therapy selection.^57^ However, based on limited evidence, it is reiterated that the presence of IDCP should be reported even when incorporated in grading, as its presence may provide independent management information (IDCP may confer resistance to radiation and androgen deprivation therapy), and for patients with intermediate‐risk PCa, testing for germline or somatic mutations detected by next‐generation sequencing performed on core biopsies containing IDCP for DNA repair gene mutations may provide information useful for managing the patient.^50^, ^51^, ^58^ The current statement of excluding patients with GP3 + IDCP (GG1 + IDCP scenario) in active surveillance is aligned with the latest clinical recommendations driven by the acceptance of IDCP as an adverse prognostic factor.^51^, ^52^

The new recommendation of incorporating IDCP in grading (per above) when associated with GG1 PCa could potentially result in the ‘disappearance’ of cases diagnosed as Gleason pattern 3 (GG1) + IDCP, as these cases would be graded as GG2–4, depending on the morphology and extent of IDCP. Regardless, the presence of IDCP in these cases must be documented even after it is incorporated in the GG. This consequence may explain why 30% of experts voted against this measure, given the paucity of long‐term follow‐up data in this rare (GG1 + IDCP) scenario. Incorporation of IDCP into the grade may lead to a heterogeneous GG2–4. For example, both GG3 + IDCP and GG1+ > 50% IDCP will now be rendered a grade of GG3. A recent large study on PCa in NBX showed GG3 to have a heterogeneous risk, and unfavourable histology, such as IDCP, was significantly associated with the risk of recurrence.^59^ Because of the rarity and challenges in the management of cases where IDCP has been included with GP3 PCa to determine the final grade, identification of these cases remains essential and should be commented upon, and it was strongly advised by experts that these patients should be referred to larger centres for diagnostic confirmation and management. Use of mpMRI has resulted in lower rates of NBX and detection of clinically insignificant cancer, and increased detection of clinically significant cancer.^60^, ^61^, ^62^ It is the opinion of expert urologic pathologists that management of NBXs with IDCP in isolation or associated with invasive PCa should factor in MRI findings. The role of advanced imaging continues to expand and is established in PCa management; however, there is still variability in interpretation and imaging quality that may limit clinical decisions solely dependent on imaging.

### Future Directions and Research Gaps in IDCP/AIP

The Boston USCAP GUPS/ISUP consultation (Supplementary Table S1) highlighted several areas of critical importance that need to be addressed through future studies, where practice remains heterogeneous, and evidence is incomplete. Specifically, future efforts that target refinement and recalibration of the Guo & Epstein IDCP criteria pertinent to current practice, help improve awareness of borderline AIPs, the development of novel biomarkers and artificial intelligence algorithms that can distinguish AIP from HGPIN, help understand the impact of incorporating IDCP into grading and risk stratification models, specifically, whether cribriform IDCP has different prognostic and/or therapeutic implications than cribriform PCa, help understand the role of mpMRI in the diagnosis and management of IDCP/AIP, the development of radiomic signatures and imaging characteristics that can help in improving the sensitivity and specificity of IDCP detection in NBX and finally, given reported links between IDC‐P/cribriform morphology and BRCA2/HRR alterations, guidelines for germline and/or somatic testing, and their management implications, are needed.

In conclusion, by employing a structured, transparent Delphi process—with iterative discussion, predefined consensus thresholds, and open community input—we were able to reconcile previously divergent guidance into a single, implementable framework. As co‐chairs of this consultation (RBS and GK), we hope that this joint effort provides a pragmatic pathway for future alignment between GUPS and ISUP, and this method can serve as a blueprint for forthcoming joint statements on topics of broad clinical relevance, helping to minimise avoidable variation in diagnostic practice across societies. A summary of key recommendations from this consultation for the diagnosis, terminology, grading, reporting, and management of IDCP and AIP is provided in Table 3. Their adoption should reduce interobserver variation, facilitate consistent communication with clinicians, improve patient management, and foster further research in this area.

**Table 3:** Summary of key practice recommendations of IDCP consultation

1	The diagnosis of IDCP should be based on the modified Guo and Epstein criteria in both biopsy and resections. In rare cases, marked (bizarre/pleomorphic) intraductal cytological atypia alone is sufficient to diagnose IDCP (after careful exclusion of mimics such as radiation atypia and pagetoid spread of high‐grade urothelial carcinoma)
2	AIP in which IDCP is favoured but the criteria are not fully met should be reported as “*AIP, suspicious for IDCP*”. The diagnosis of cribriform high‐grade PIN should not be made. Such lesions should be reported as “*AIP, suspicious for IDCP*”. ERG and PTEN immunohistochemistry, if available, could be helpful when HGPIN is in the differential diagnosis
3	Pure or isolated IDCP without an associated invasive PCa and IDCP component that is spatially distinct from invasive PCa should not be included in grading. Judicious use of basal cell markers to confirm the diagnosis in these settings is recommended
4	AIP, suspicious for IDCP, should not be incorporated in the grading
5	IDCP component should be incorporated in the grading when associated with invasive PCa, regardless of Grade Group
6	Grading of IDCP component should be based on its morphology: cribriform IDCP as Gleason pattern (GP) 4, solid and comedonecrosis IDCP as GP5
7	Active surveillance is not appropriate for patients with GP3 associated with IDCP. Due to the rarity of such a situation, a second opinion by a dedicated GU pathologist and discussion in a multidisciplinary setting is recommended, and the management decision should also factor in the multiparametric MRI result

## Author contributions

RBS and GK contributed to the study concept and oversight; RBS, MV, MZ, GP, and GK contributed to the study design, development of the survey and methodology, execution of the project, and manuscript writing; All authors contributed to multiple rounds of surveys, consultations, and have read and approved the final paper.

## Conflict of interests

No authors have declared a conflict of interest in this study.

## Supporting information


**Table S1.** IDCP consultation Boston meeting participants.

## Data Availability

Data are available for bona fide researchers who request them from the corresponding authors.
