# Supplementary material for: The Genitourinary Pathology Society and International Society of Urological Pathology Joint Expert Consultation Recommendations on intraductal carcinoma of the prostate
Source: Histopathology. 2025 Dec 12;88(1):8–23. doi: 10.1111/his.70046 (PMC12700062; doi:10.1111/his.70046)
Supplement: Supplementary file 1 — Table S1. IDCP consultation Boston meeting participants. [file HIS-88-8-s001.docx]

**Table S1.** IDCP consultation Boston meeting participants

| **Name** | **Country** | **Name** | **Country** |
| --- | --- | --- | --- |
| Imen Abbes ᴕ | Tunisia | Ali Lowe ᴕ | United States |
| Andres Acosta | United States | Fiona Maclean | Australia |
| Adebowale Adeniran ᴕ | United States | Miranda Machacek | United States |
| Samita Agarwal ᴕ | United Kingdom | Cristina Magi-Galluzzi # | United States |
| Mohamed Ahmed ᴕ | United Kingdom | Alopa Malaviya ᴕ | United Kingdom |
| Mahmut Akgul | United States | Varsha Manucha | United States |
| Roula Albadine ᴕ | Canada | Romulo Mattedi ᴕ | Brazil |
| Hikmat Al-Ahmadie ᴕ | United States | Teresa McHale ᴕ | Ireland |
| Termina Ali ᴕ | United States | Ana Rosa González Medina ᴕ | Spain |
| Fawaz Almutairi ᴕ | Saudi Arabia | Rohit Mehra | United States |
| Mahul Amin # | United States | Santosh Menon | India |
| Chantal Atallah | Canada | Hiroshi Miyamoto # | United States |
| Daniel Athanazio | Brazil | Abubakr Mohamed ᴕ | United Kingdom |
| Sahar Azad ᴕ | United Kingdom | Waria Mohamid ᴕ | United Kingdom |
| Sha Bai ᴕ | United States | Renn Montgomery ᴕ | Australia |
| Serdar Balci ᴕ | Turkey | Mariana Morini ᴕ | Brazil |
| Ashish Bansal ᴕ | United Kingdom | Subramanian Murugesan ᴕ | India |
| Marc Barry | United States | Sri Nagarajan ᴕ | United Kingdom |
| Dilek Baydar | Turkey | Gabriella Nesi | Italy |
| Busra Yaprak Bayrak | Turkey | Jane Nguyen # | United States |
| Marit Bernhardt ᴕ | Germany | Maya Nourieh ᴕ | France |
| Chew Bee See ᴕ | Malaysia | Marie O’Donnell ᴕ | United Kingdom |
| Shubha Bellur ᴕ | Canada | Gladell Paner +# | United States |
| Beatriz Bordon ᴕ | United States | David Parada ᴕ | Spain |
| Daniel Berney # | United Kingdom | Jenish Patel ᴕ | United Kingdom |
| Stephania Bezerra | Brazil | Constantina Petraki | Greece |
| Ruth Birbe | United States | Maria Picken | United States |
| Fadi Brimo ᴕ | Canada | Flavia Guzman Pineda ᴕ | Canada |
| Qi Cai ᴕ | United States | Maria JR Pinto ᴕ | United Kingdom |
| Emily Chan ᴕ | United States | Kristyna Pivovarcikova | Czech Republic |
| Jie-Fu Chen | United States | Miruna Popescu | Germany |
| Ying-Bei Chen | United States | Preeti Rai ᴕ | United Kingdom |
| Liang Cheng # | United States | Juan David Cuartas Ramirez ᴕ | Colombia |
| Yong Mee Cho ᴕ | South Korea | Marzena Ratynska ᴕ | United Kingdom |
| Vaibhav Chumbalkar | United States | Maria Rosaria Raspollini | Italy |
| Adam Cole | United States | Dong Ren | United States |
| Fernanda Cordeiro-Rudnisky ᴕ | United States | Jordan Reynolds | United States |
| Kristine Cornejo | United States | Brian Robinson | United States |
| Chunhua Cui ᴕ | United States | Sahar Rozza ᴕ | Egypt |
| Isabela da Cunha | Brazil | Paromita Roy | India |
| Pamela de Leon ᴕ | Guatemala | Tapan Saikia ᴕ | India |
| Darius Dasevičius ᴕ | Lithuania | Jonathan Salmond ᴕ | United Kingdom |
| Kaushik Dasgupta ᴕ | United Kingdom | Iryna Samarska ᴕ | Netherlands |
| Warick Delprado | Australia | Mohammed Sami Saeed ᴕ | United Kingdom |
| Angelo DeMarzo | United States | Angela Sanguino ᴕ | United States |
| Fang-Ming Deng # | United States | P Anna Matilda Saparamadu ᴕ | United Kingdom |
| Sabina Desar ᴕ | United States | Judy Sarungbam | United States |
| Jasreman Dhillon ᴕ | United States | Anna Scherping ᴕ | Germany |
| Duy Doan ᴕ | United States | Rajal Shah *+# | United States |
| Michelle Downes # | Canada | Svetlana Shalygina ᴕ | Spain |
| Brinza Dumitru ᴕ | Moldova | Steven Shen | United States |
| Scott Eggener ^ | United States | Salome Shukla ᴕ | India |
| Behfar Ehdaie ^ | United States | Stephanie Siegmund | United States |
| Carla Ellis | United States | Jeff Simko | United States |
| Amira Elsherif ᴕ | United Kingdom | Steven Smith | United States |
| Jonathan Epstein # | United States | Luis Sosa | Guatemala |
| Andrew Evans # | Canada | John Srigley | Canada |
| Sara Falzarano | United States | Elena Stoica-Mustafa ᴕ | Romania |
| Michael Feely ᴕ | United States | Yue Sun | United States |
| Samson Fine # | United States | Martina Susani ᴕ | Austria |
| Eddie Fridman ᴕ | Israel | Sueli Suzigan | Brazil |
| Paul Friedman | United States | Fawzia Tahir ᴕ | United Kingdom |
| Liying Fu ᴕ | United States | Monia Tangour ᴕ | Tunisia |
| Laurence Galea ᴕ | Australia | Sahrah Tawil ᴕ | Malaysia |
| Jatin Gandhi ᴕ | United States | James Thackeray | United States |
| Marilia Germanos | Brazil | Phataraporn Thorson ᴕ | United States |
| Giovanna Giannico | United States | Satish Tickoo | United States |
| Francesca Giunchi ᴕ | Italy | Levent Trabzoulu | United States |
| Neriman Gokden ᴕ | United States | Patricia Troncoso ᴕ | United States |
| Fatma Gundogdu ᴕ | United Kingdom | Maria Tretiakova | United States |
| Robert Goulart | United States | Dominque Trudel | Canada |
| Nancy Greenland # | United States | Larry True | United States |
| Raluca Grigorescu ᴕ | United Kingdom | Toyonori Tsuzuki # | Japan |
| Charles Guo # | United States | Monika Ulamec ᴕ | Croatia |
| Nadezda Gut | Germany | Saleem Umar ᴕ | United States |
| Aiman Haider ᴕ | United Kingdom | Nicolò Vianini ᴕ | Italy |
| Trine Hallager ᴕ | Denmark | Jacob Valk | United States |
| Bo Han # | China | Theodorus van der Kwast # | Canada |
| Lara Harik | United States | Sumiti Vanjani ᴕ | United Kingdom |
| Oudai Hassan | United States | Murali Varma +# | United Kingdom |
| Loren Herrera Hernandez | United States | Geert van Leenders # | Netherlands |
| Michelle Hirsch # | United States | Sangeeta Verma ᴕ | United Kingdom |
| Christina Hulsbergen-van de Kaa | Netherlands | YJ Wang ᴕ | Taiwan |
| John Hunt | United States | Anne Warren ᴕ | United Kingdom |
| Nuzhat Husain | India | Thomas Wheeler ᴕ | United States |
| Kenneth Iczkowski # | United States | Sean Williamson # | United States |
| Muhammad Idrees ᴕ | United States | Sara Wobker # | United States |
| Payal Kapur | United States | Kirk Wojno | United States |
| Beerinder Pal Karir ᴕ | United States | Chin-Lee Wu # | United States |
| Riham Katkhuda ᴕ | United States | Kin-Chung Wu ᴕ | United Kingdom |
| James Kench # | Australia | Linjie Xiong ᴕ | United States |
| Meena Swar Khadka ᴕ | United Kingdom | Yiqin Xiong ᴕ | United States |
| Francisca Khani | United States | Bo Xu ᴕ | United States |
| Okan Kilic | United States | Lavinia Y ᴕ | Australia |
| Matthias Koslowski ᴕ | United Kingdom | Anglita Yantisetiasti ᴕ | Indonesia |
| Glen Kristiansen *+# | Germany | Ximing Yang # | United States |
| Jung Woo Kwon | United States | Oksana Yaskiv | United States |
| Sana Jadallah ᴕ | United States | Huihui Ye ᴕ | United States |
| Jacub Khzouz ᴕ | Jordan | Yunshin Yeh | United States |
| Priti Lal ᴕ | United States | Gurbet Yokuʂ ᴕ | Turkey |
| HyunJung Lee ᴕ | South Korea | Miao Zhang | United States |
| Subodh Lele | United States | Xiaotun Zhang | United States |
| Maria Sarah Lenon ᴕ | Philippines | Ting Zhao | United States |
| Duane Leonard ᴕ | United States | Yayuan Zhao ᴕ | Canada |
| Claudio Lewin | Argentina | Lan Zheng ᴕ | United States |
| Jianhong Li ᴕ | United States | Ming Zhou +# | United States |
| Massimo Loda | United States | Yang Zong ᴕ | United States |
| Tamara Lotan # | United States | Debra Zynger ᴕ | United States |

* Chair; + Core Group; # Pathologist Consultant; ^Urologist Consultant; ᴕ Online participants for at >1 hour
